# Path of infectious diseases in Brazil in the last 50 years: an ongoing challenge

**DOI:** 10.1590/S1518-8787.2016050000232

**Published:** 2016-11-24

**Authors:** Eliseu Alves Waldman, Ana Paula Sayuri Sato

**Affiliations:** I Departamento de Epidemiologia. Faculdade de Saúde Pública. Universidade de São Paulo. São Paulo, SP, Brasil

**Keywords:** Communicable Diseases, Communicable Diseases, Emerging, Public Health, Scientific and Technical Publications, Historical Article

## Abstract

In this article, we comment on the main features of infectious diseases in Brazil in the last 50 years, highlighting how much of this path *Revista de Saúde Pública* could portray. From 1967 to 2016, 1,335 articles focusing on infectious diseases were published in *Revista de Saúde Pública*. Although the proportion of articles on the topic have decreased from about 50.0% to 15.0%, its notability remained and reflected the growing complexity of the research required for its control. It is noteworthy that studies design and analysis strategies progressively became more sophisticated, following the great development of epidemiology in Brazil in the recent decades. Thus, the journal has followed the success of public health interventions that permitted to control or eliminate numerous infectious diseases – which were responsible, in the past, for high rates of morbidity and mortality –, and also followed the reemergence of diseases already controlled and the emergence of until then unknown diseases, with a strong impact on the Brazilian population, establishing a little predictable and very challenging path.

## RESUMO

Neste artigo, comentamos as principais características das doenças infecciosas no Brasil, nos últimos 50 anos, destacando o quanto a Revista de Saúde Pública conseguiu capturar essa trajetória. De 1967 a 2016, foram publicados 1.335 artigos na Revista de Saúde Pública com foco em doenças infecciosas. Ainda que a proporção de artigos sobre esse tema tenha declinado de cerca de 50,0% para 15,0%, seu destaque se manteve e refletiu a crescente complexidade das pesquisas necessárias para o seu controle. Nota-se que os desenhos dos estudos e as estratégias de análise ganharam progressivamente maior sofisticação, acompanhando o grande desenvolvimento da epidemiologia no Brasil, nas últimas décadas. Assim, foi registrado não apenas o sucesso de intervenções de saúde pública que permitiram o controle ou a eliminação de inúmeras doenças infecciosas responsáveis, no passado, por elevadas taxas de morbimortalidade, como também a reemergência de males já controlados e o surgimento de doenças até então desconhecidas, com forte impacto na população brasileira, desenhando uma trajetória pouco previsível e muito desafiadora.

## INTRODUCTION

The social, economic, and demographic transformations that took place in the last 50 years have been determining factors of significant changes in the morbidity and mortality patterns around the globe. In the context of such transformations, the expansion of sanitation coverage, improvement of housing conditions, and introduction of new health technologies, particularly vaccines and antibiotics, were decisive for the rapid decline in the magnitude of infectious diseases^25^ ^,^ ^99^.

This new scenario has led, in the 1960s and 1970s, to the optimistic perception that this group of diseases would lose relevance in public health as economic development and access to better living conditions could be widely achieved by most countries^57^.

However, the facts contradicted such expectations and what we saw was the acceleration of the emergence and reemergence process of infectious diseases from the end of the 20th century on, keeping them on the list of priorities of the Public Health Global Agenda^58^.

This path of continuous and often unexpected changes assumed a global aspect, but with different speed and intensity in the countries. For its continental dimensions, large population, pronounced regional contrasts, and for having presented in the last half-century fast and accentuated changes in its socioeconomic, demographic, and health indicators, Brazil is a case of special interest^22^ ^,^ ^119^ ^,^ ^120^ ^,^ ^131^.

Celebrating the 50 years of *Revista de Saúde Pública* (RSP), we developed this study with the objective of describing and commenting on the main features of infectious diseases in Brazil, from 1967 to 2016, highlighting those that have attained greater relevance in the public health agenda and pointing out the most striking aspects recorded in the articles published on the topic by RSP.

With this purpose, we describe the behavior of infectious diseases in Brazil in the period, and comment on the most relevant aspects portrayed by RSP, grouping these diseases into three categories: (i) diseases with strong decline tendency; (ii) diseases with mild decline tendency; (iii) diseases that have assumed emerging and reemerging quality.

For this review to be the most complete possible, we surveyed, year after year, all the keywords related to the topic linked to articles published by RSP. Then, we searched SciELO database, selecting all texts focusing on the topic of interest. In this process, we identified 1,335 articles and selected 146 that highlighted relevant moments of the path of infectious diseases in Brazil.

### Brief Report on the path of Infectious Diseases in Brazil (1967-2016)

Over the past five decades, Brazil has suffered profound transformations – its population more than doubled, reaching 200 million inhabitants, and urbanization has increased (nowadays, 83.0% of its inhabitants live in cities), in addition to the fast process of population aging. Income *per capita* and education increased and, in turn, child mortality significantly decreased, from 117 to 16 deaths per 1,000 live birth^22^ ^,^ ^119^ ^,^ ^120^ ^,^ ^131^. The Country’s Human Development Index is currently classified as high, scoring 0.755, contrasting with 0.545 in 1980.

Consistent with this new scenario, infectious diseases lose their relative importance because the ratio of deaths associate with them decreased from 35.0% to about 5.0% nowadays^22^ ^,^ ^156^ ^,^ ^172^. This favorable tendency was due especially to the drastic decrease of deaths by diarrhea and vaccine-preventable diseases^118^ ^,^ ^178^ ^,^ ^181^.

Contrary to this tendency, the Country was plagued by major epidemics, such as meningococcal disease in the 1970s (possibly the most serious epidemic outside the sub-Saharan region, in Africa, throughout the 20th century), an event well described on the pages of RSP^14^ ^,^ ^90^. Brazil was also heavily affected by the emergence of HIV^23^ ^,^ ^130^. In addition, we have seen the emergence of three arboviruses: Rocio virus encephalitis causes a serious epidemic in the region of Vale do Rio Ribeira, SP, in the 1970s^91^ ^,^ ^163^ and, more recently, the emergence of Chikungunya^13^ ^,^ ^55^ and Zika^36^ ^,^ ^89^ ^,^ ^104^ viruses.

We also observe the reemergence of diseases considered controlled or eliminated, such as dengue in the 1980s, cause for growing concern by the increased incidence of its serious manifestations and the mortality associated with it^136^ ^,^ ^154^ ^,^ ^181^, and trachoma, which was highlighted in the past as a major cause for blindness^72^ and which, from the 1980s, is again reported^34^ ^,^ ^51^ ^,^ ^106^ ^,^ ^108^. The reemergence of cholera also caused surprise in the 1990s, after a century of its absence in the Americas^78^ ^,^ ^179^.

### Infectious Diseases in Revista de Saúde Pública

Articles published by RSP in its half century of existence allow monitoring not only the epidemiological transition in the Country, but also feeling the consolidation of the multidisciplinary quality of public health. During this period, the proportion of articles focusing on infectious diseases decreases from about 50.0% to 15.0%, without, however, losing its prominence. Such texts reflect the increased complexity of public health in the Country and the decentralization of research in this field of knowledge, which is consolidated in all regions of Brazil, along with dissemination of the graduate programs in Public Health. We note in these articles studies with progressively more complex designs and more robust analysis strategies, following the development of epidemiology in Brazil in the last decades^21^ ^,^ ^85^.

This downward path of infectious diseases – paradoxically, full of potentially serious situations –, was recorded in articles published by RSP. The multidisciplinary quality of the journal offers its readers subsidies to understand the complex interaction of factors involved in the behavior of infectious diseases, which is often unpredictable. This characteristic allows a single infectious disease presenting, at different times in the same population, profiles of distinctive behaviors, which often bring great challenges to society and, especially, to health professionals and researchers, professionals whose mission is to protect and promote the population’s health.

### I. Infectious Diseases with Strong Decline Tendency

#### Rural Endemic Diseases

In the 1960s and 1970s, infectious diseases were highlighted specially on the public health priority agenda; among them, the so-called rural endemic diseases. Samuel Barnsley Pessoa^134^ (1963), in one of his classic works, commented on health conditions of the Brazilian rural population, noting the high prevalence of malnutrition and chronic hunger, bad housing and hygiene conditions, and the complete lack of sanitation to which such population was exposed. Under these conditions, rural areas, which in the 1960s comprised approximately 50.0% of the Country’s population, featured hyperendemic levels of several parasitic diseases, among which outstood masonic schistosomiasis, Chagas disease, and malaria.

The rapid industrialization of the Country and the resulting urbanization, economic development, expansion of education at all levels, and the establishment of the Brazilian Unified Health System transformed, especially over the past three decades, the Brazilian health framework, creating conditions for eliminating rural endemic diseases of the public health agenda of priorities in the Country^120^ ^,^ ^131^ ^,^ ^181^.

Malaria has its history well documented on the pages of RSP, with the publication of dozens of articles on the topic. In the 1960s and 1970s, the highlight was the discussion of the performance of strategies to control this endemic disease in several Country regions^12^ ^,^ ^47^. In the 1980s, with favorable results in the control of malaria, which practically restricted its endemic transmission to the Legal Amazon region, new challenges were highlighted, such as resistance to drugs^3^ ^,^ ^60^ and the impact of development projects on the Amazon, due to the risk of reintroducing the disease in regions where it was already controlled^174^. More recently, following the discussion of new challenges for controlling diseases transmitted by vectors, interesting studies have been published on climate change and malaria transmission^184^ ^,^ ^185^.

Chagas disease also outstood on RSP pages, with approximately 100 articles published on the topic. Of these articles, we have classic texts by Forattini on the biology of Triatominae^63^ ^-^ ^68^. Studies on the burden of Chagas disease in different regions of the Country^40^ ^,^ ^155^ ^,^ ^176^, control strategies^139^ ^-^ ^141^, and alternative transmission types of Chagas disease^175^ are also present, as well as the disease social impact and, more recently, an interesting article on the 100th anniversary of the Chagas disease description^6^.

Mansonic schistosomiasis was also widely covered by RSP, with 75 articles addressing various aspects of this endemic disease. On its pages, we find the most important records of the spread of schistosomiasis in Sao Paulo^11^ ^,^ ^110^ ^,^ ^132^ ^,^ ^138^, as well as relevant research on the biology of its intermediate host^101^, studies focusing on biological control of planorbids^86^ and on the planorbid chart of São Paulo state^170^ ^,^ ^171^. It is worth highlighting two articles that explore aspects little addressed in our literature: the study by Silva^157^ (1985) described the disease expansion process in São Paulo and defends the thesis that the urbanization pattern would have been more relevant than the migration in the schistosomiasis expansion; the article written by Barreto^20^ (1987), in turn, discussed the importance of studies on causal and predictor factors of this parasitosis for the development of intervention strategies.

#### Acute Diarrhea

In the 1960s, urban life conditions in Brazil were very unfavorable, a situation exacerbated by the intense process of migration from the countryside to the cities, which was the responsible for the rapid and disorderly growth of our cities and accentuated bad housing conditions and lack of basic sanitation. As a result, child mortality was very high, mainly caused by diarrhea, even in the richest capitals of the Country^102^ ^,^ ^118^. Transformations faced by the Country in the following decades allowed such scenario to be radically changed, making diarrhea a little relevant cause for child morbidity and mortality^128^.

The path of acute diarrhea is well described by RSP, not only quantifying the burden of disease and its reflections on child mortality^27^ ^,^ ^28^ ^,^ ^75^ ^,^ ^118^ ^,^ ^128^, but also addressing interventions such as oral rehydration^76^, and the change in its seasonality^95^ ^,^ ^178^ and etiologic pattern^37^. Moreover, pioneer articles about rotavirus outstand^38^.

#### Vaccine-Preventable Diseases

In the 1960s and 1970s, acute diarrhea was not the only disease afflicting the child population in Brazil. Vaccine-preventable diseases were endemic and responsible for high rates of morbidity and mortality. Measles was considered the leading cause of death among children aged from one to four years in major cities from different regions of Country^137^. Poliomyelitis was epidemic, leaving many individuals with motor sequelae, often aggravated by late manifestations (post polio syndrome) that worsen the quality of life^46^ ^,^ ^152^.

The creation of the successful *Programa Nacional de Imunizações* (PNI – National Immunization Program) in 1973 drastically reduced morbidity and mortality by vaccine-preventable diseases, including the elimination of poliomyelitis in 1989 and the practically absence of sustained measles transmission in the whole Country since 2001^181^, as well as the eradication of smallpox, certified in 1980^69^ ^,^ ^70^.

With over a hundred articles on this group of diseases, RSP recorded in a classic study by Barbosa^15^ (1969) the great epidemics of poliomyelitis in the 1950s and 1960s, which occurred in the period prior to the introduction of the vaccine. It also published some of the first studies on seroprevalence of poliovirus antibodies^16^ ^,^ ^17^ ^,^ ^177^ and relevant information for the development of vaccination strategies. Moreover, it described the impact of mass vaccination campaigns and the higher seroconversion after three doses of trivalent polio vaccine obtained by this strategy, if compared with the exclusive application of regular vaccination^18^. More recently, in the context of the final stages of Global Polio Eradication Initiative, we had the publication of an interesting study about the cost-effectiveness of the inactivated vaccine against poliomyelitis^149^.

Measles and rubella were also object of several publications that aimed to better know their epidemiological aspects, including studies on seroprevalence^31^ ^,^ ^41^ ^,^ ^135^, and the operational aspects regarding the conservation of measles vaccine^114^. Recently, when measles no longer showed sustained transmission in a large part of the Country, RSP registered a disease outbreak from an imported case, in the metropolitan area of São Paulo^59^.

We also find articles that contribute to better understand the vaccine coverage achieved by PNI, showing a significant decrease in disparities in different social strata^107^ ^,^ ^116^, and studies that investigate factors associated with non-adherence to vaccination^159^, which certainly contributed to the improvement of vaccination strategies. In addition, RSP has published articles focusing on new strategies for vaccination against whooping cough, before the possible disease reemergence in Brazil^74^.

The recognized success of PNI and the significant expansion of the vaccines included in the national calendar of immunizations make this program more complex, creating new challenges such as maintaining the high vaccine coverage, equity in access, and safety. Several studies address these issues: methods used to assess the impact of vaccination programs^150^; surveillance strategies focusing on the evaluation of vaccination safety^73^ ^,^ ^180^, and discussions about the incorporation of new information technologies for real-time monitoring of vaccine coverage, including micro areas, and the identification of vaccine lots with greater reactogenicity^151^.

## II. Infectious Diseases with mild Decline Tendency

### Tuberculosis and Hanseniasis

Tuberculosis (TB) was covered by 130 articles in RSP, highlighting the main points of its path in Brazil over the last 50 years. In turn, hanseniasis was addressed by 30 articles. Commonly, both diseases are strongly related to poverty and poor living conditions^50^. Furthermore, they presented a mild but consistent decline in the period and suffered, albeit with different intensities, the impact of the emergence of AIDS^8^ ^,^ ^181^. We also highlight that the influence of AIDS on the hanseniasis behavior was little studied^112^.

In the 1970s, we had the dissemination of the results of an important tuberculin investigation with students, developed by Certain et al.^44^ (1972), in addition to relevant articles about planning and evaluation techniques for TB control activities in health services^9^ ^,^ ^10^ and new treatment schemes^33^. We also found texts on robust methods of risk estimation of tubercular infection^144^ ^,^ ^145^, an indispensable support to the improvement of TB control strategies. We also highlight an early case-control study published in Brazil, which investigated the TB risk factors^39^.

In the 1980s, we had studies that analyzed the effectiveness of short term treatment schemes newly introduced^26^ at that time and about the low efficiency and health risks of chest photofluorography (or roentgenphotography) as screening strategy for discovering new cases^4^ ^,^ ^81^. From the 1990s on, studies started to address the importance of TB/HIV coinfection^97^ ^,^ ^127^ ^,^ ^148^, as well as the differences in the TB incidence for older age groups ^45^.

More recently, analyses on the high social impact of TB and estimates of its treatment costs^49^, new approaches to its surveillance^161^, and one pioneering study that analyzed the effectiveness of revaccination strategy of adolescents with BCG^35^ were published.

In the last decade, RSP published current texts on the topic, and those on the challenges and perspectives of TB control strategies in Brazil^19^ ^,^ ^142^ stand out. These studies analyzed the burden of disease, its tendency, and current profile^29^ ^,^ ^30^ as well as the drug resistance situation^52^. Another aspect that deserved emphasis in recent years has been the TB behavior in vulnerable populations^77^ ^,^ ^126^ ^,^ ^129^, besides special emphasis on high mortality and surveillance strategies for this outcome^103^ ^,^ ^153^.

Regarding hanseniasis, we highlight articles that estimate the burden of disease in different regions of the Country^2^ ^,^ ^105^ ^,^ ^124^ and those describing the main aspects of its behavior in high-endemicity areas^123^. We have two texts that deserve special highlight for their historical importance: the article of Guimarães^87^, which analyzes the integration of care of patients with hanseniasis in general hospitals, and the comment of Rotberg^143^ on the pathogenic theory of Hansen’s disease.

## III. Emerging and Reemerging Diseases

Despite the recent highlight, the emergence of infectious diseases is not a new phenomenon. Among the most cited old records about the topic, we have a pandemic of bubonic plague at the end of Middle Age^100^ ^,^ ^122^. Some factors give to this type of event great importance in public health: the unpredictability, the possibility of strong impact on demographics caused by the rapid elevation of mortality rates, the impact on the economy of affected populations, and the potential to assume a pandemic behavior^58^ ^,^ ^89^ ^,^ ^122^ ^,^ ^158^.

In the last two decades, the increase of the frequency and speed with which these events have occurred is associated with many factors, including globalization, increased international exchanges, and the intensive use of mass air and urban transportation, along with the fast population growth and the accelerated urbanization process that has recently intensified in developing countries with large populational contingent^32^ ^,^ ^57^.

The emergence and reemergence of infectious diseases in Brazil have often been interpreted as resulting from worsening living conditions of the Brazilian people, especially considering the urban infrastructure of major cities, which would mean a retrocession back to the situation lived by the Country in the early 20th century. However, this interpretation is mistaken, since these emergence and reemergence cycles comprise characteristics of this group of diseases, even though the lack of urban infrastructure is an aggravating feature.

### Reemerging Diseases

Among reemerging diseases of bacterial etiology reported in Brazil, in the last 50 years, we have the Brazilian purpuric fever that in the 1980s spread to states in the South, Southeast, and Midwest regions. It deserved special attention at the time for affecting 10-year-old children, with high lethality (about 70.0%), and the potential risk to reach large urban centers. It was an unknown disease at that time, and its etiologic agent – *Haemophilus influenzae biogrup aegyptius* – was identified after exhaustive research^88^. RSP has published a relevant study to elucidate its etiology^96^. For little-known reasons, this disease is no longer reported in the Country since 1993; however, in 2007 five suspected cases of the disease, although unconfirmed, were reported in Ilha do Marajó, in the state of Pará, Brazil^147^.

Cholera also reemerged in 1991, entering Brazil by the Amazon region, at the Colombia and Peru borders. The disease affected more intensely small towns and some capitals of the North and Northeast regions, especially populations without access to basic sanitation. Studies published by RSP suggest that the impact of cholera mortality on these regions would have been underestimated because of faults in information systems^78^ ^,^ ^79^ ^,^ ^133^.

The reemergence of trachoma in the 1990s has been registered in RSP by the publication of research with students in the Southeast Region^98^ ^,^ ^113^ and a recent national investigation, which shows higher prevalence in rural areas and among children under five years of age, although severe forms are rare^106^.

Visceral leishmaniasis also stands out – the disease was the subject of 30 articles. Its relevance is due to rapid urbanization and expansion of its transmission area, and the high lethality (approximately 10.0%)^183^. The path of visceral leishmaniasis in recent decades is well described in the pages of RSP^48^ ^,^ ^93^ as well as the difficulties for its control. Such difficulties are due to the fact the diagnosis and early treatment of human cases are essential to prevent deaths, but are ineffective to prevent the transmission. On the other hand, its control by interventions focusing on reservoirs has faced obstacles such as those commented by Werneck^183^ in detail.

The reemergence of yellow fever, which currently gains international importance, was prominently reported in RSP. Brazil has two wide areas with potential risk of transmission: one is enzootic and extends through forests of the Amazon and Midwestern Regions; the other, of epizootic behavior, covers the states of Rio Grande do Sul, Santa Catarina, Paraná, São Paulo, and Minas Gerais^92^ ^,^ ^169^. In turn, the wide dispersal of *Aedes aegypti* in the Country is a warning for potential urban outbreaks^165^ ^,^ ^169^.

Historically, Brazil has regular cycles of increased cases of yellow fever among human beings every five years, suggesting a predictable behavior. However, since the beginning of this century, we see smaller and irregular intervals between the peaks of incidence of the disease and, more worrying, the recent occurrence of outbreaks in the states of São Paulo and Rio Grande do Sul, which have been free of the disease for decades. The risks are not negligible, especially considering the occurrence of two epidemics of urban yellow fever in bordering countries: in Bolivia, in 1999, and in Paraguay, in 2007^165^ ^,^ ^169^.

The report in China, in 2016, of several imported cases of yellow fever from an ongoing epidemic in Angola – urban type of the disease –, illustrates the potential magnitude of the problem, considering that part of the Chinese territory is endemic for dengue and that international vaccine stocks could be insufficient to deal with a situation of international emergency^182^.

Among infectious diseases that reemerged in Brazil in the last 50 years, dengue was the one assuming greater importance. Since the reintroduction of *Aedes aegypti* in the Country, in 1976, it was only a matter of time for the emergence of the first epidemics of dengue fever in Brazil, following the vector dispersal. This happened in 1981 with the first outbreak in Roraima^164^. Since the early 1990s, we have an endemic circulation of three serotypes (DENV-1, DENV-2, and DENV-3), situation that became more worrisome, in 2010, with the reintroduction of DENV-4^162^ ^,^ ^166^.

In 2007, *Aedes aegypti* was already found in approximately 70.0% of Brazilian municipalities and dengue fever occurred in half of them, with significant presence in medium and small cities. In some capitals, seroprevalence was over 65.0%^166^. Another important aspect observed over the past 10 years is the proportional increase of cases with people aged under 15 years, with the increase in severe forms of the disease and, consequently, in hospitalizations and deaths. In some regions of the Country, these severe forms have affected more intensely younger age groups^166^. Such data place this arbovirus among major public health problems in the Country and also suggest the possible aggravation of this situation.

The RSP marked important moments of the path of dengue in Brazil, with articles describing a history of *Aedes aegypti* control programs in Brazil^154^, commenting on the introduction of *Aedes albopictus* in the Country and its potential impacts^71^, the first epidemics in medium cities of the Southeast region^136^, important studies on the vector behavior^111^, seroprevalence surveys in Brazilian capitals^168^ as well as socioeconomic characteristics and their relation with the vector density and the occurrence of dengue^61^ ^,^ ^117^. Finally, in recent years, we have an article commenting on the obstacles and challenges to the development of a vaccine against dengue^1^.

### Emerging Diseases

Among emerging diseases, the one that determined the greatest impact on Brazil was certainly AIDS, topic explored in about 200 RSP articles, including four special issues. The first study, published in 1989, showed HIV seroprevalence (0.07%) from tracking tests in blood banks in Goiânia (GO), emphasizing the risk of transmission by blood transfusion and the need for control^7^.

Important aspects of the path of AIDS epidemic were portrayed by RSP. The behavior of the disease has changed a lot in this period due to the evolution of knowledge and therapeutic and diagnostic resources. Studies show a tendency of stabilization or decline in the incidence and mortality from AIDS from 1996, due to the introduction of highly active antiretroviral therapy, albeit in a heterogeneous way in the Country^54^ ^,^ ^82^. It should be noted that a disease of acute and fatal quality initially assumes features of a chronic disease with increasing incubation period and survival rate^84^. To the extent that the prevalence of HIV/AIDS increases, the demand for basic health services also increases, in addition to the demand for specialized network, either for treatment, diagnosis, or prophylaxis^125^.

Several articles showed the change of groups vulnerable to HIV infection throughout the period. Initially, the disease reached mainly male homosexuals of middle and upper-middle class, then started affecting women, and also the more needy population^56^. Topics such as social vulnerability, stigma, sexual behavior, and perceptions were covered by RSP^24^.

A major proportion of the studies published by RSP on the topic analyzed the impact of the disease on the quality of life^80^. We also highlight studies on adherence to treatment as an important challenge due to, in addition to adverse events, social and cultural factors that permeate the disease^115^. Equally important, studies on adverse events of the treatment as metabolic and morphological changes were present in RSP^5^ ^,^ ^94^.

We also found in the journal studies that describe defining opportunistic infections of AIDS. Overall, oral candidiasis appears as the most prevalent infection, followed by tuberculosis, *Pneumocystis carinii* pneumonia, and neurotoxoplasmosis^167^. Research on TB/HIV coinfection have also been frequent since the 1990s and are still today in the journal^42^ ^,^ ^146^.

Moreover, studies on AIDS with other specific populations, such as pregnant women and vertical transmission^173^, health professionals^109^, sex workers^53^, drug users^160^, and homeless population^83^ are also carried out.

An important event, but punctual and little cited currently, was the emergence, in the 1970s, of the Rocio virus, an agent transmitted by arthropods, which determined a wide encephalitis epidemic in the region of Vale do Rio Ribeira, South of the state of São Paulo, causing more than 600 cases with 10.0% of lethality and 20.0% of sequelae. This event was described in RSP by Iversson^91^ ^,^ ^92^. Other outbreaks of this arbovirus are unknown; however, the presence of Rocio virus has been reported in rural areas of the Country^62^, suggesting the risk of its reemergence.

Since 2014, Brazil has seen the emergence of two other arboviruses, Chikungunya and Zika, which, as dengue and urban yellow fever, have *Aedes aegypti* as the main vector. The Zika virus have a strong international repercussion for being associated with congenital malformations and neurological complications^36^ ^,^ ^89^. Despite being recent events, both arboviruses were covered in RSP by two very interesting and convenient articles^13^ ^,^ ^104^.

The emergence of these arboviruses exemplifies the ability of microorganisms to adapt in new host species, creating conditions for the emergence of so far unknown infectious diseases in humans, caused by agents that circulated only among animals^100^ ^,^ ^121^.

## Final Remarks

With the reintroduction of infectious diseases in the new global public health priority agenda, we note that control activities of this group of diseases are far more complex than in the past. Therefore, it is necessary, in addition to high coverage of vaccination and sanitation, an effective network of basic health services and an appropriate surveillance system.

However, without diminishing the relevance of the strategies mentioned, we perceive, since the beginning of this century, the incorporation of Internet and new information technologies, including the interconnected use of large databases, routine activities of monitoring and control of infectious diseases^43^. On the other hand, in addition to a better relation between national surveillance systems and the network of health services, we also observed the establishment of a stronger and more explicit relation between surveillance and research. This makes the knowledge production more agile, which is essential to ensure effective and appropriate interventions, making surveillance an important instrument for the continuous improvement of health services. A recent example, intensively experienced by the Country, was the emergence of Zika virus, for which was established, in response, a close relation between health services, epidemiological surveillance, research institutes, and universities, along with a strong international insertion.

This scenario brings enormous challenges to Brazilian scientific journals, especially to those that act in the field of public health, among them, RSP. Such journals are responsible – with new strategies and the incorporation of new technologies – for expanding and accelerating the diffusion of the knowledge produced, as well as strengthening their relations with researchers and professionals that work in different areas of public health and, thus, responding to the new challenges of the 21st century. The RSP, with 50 years of continuous renewal and accumulation of experience, is certainly prepared to do its share in this mission.
